# 

**Published:** 1886-05

**Authors:** 


### OUR ADVERTISERS.

SEE new advertisement of John Barry's Clinical Thermometer.

E. FOUGERA & CO., NEW YORK.—This old and well-known firm has an attractive advertisement on page 35. Refer to it and write them for samples and prices. They deal only in first-class goods.

ELECTRO-MEDICAL BATTERY CO., of Kalamazoo, Mich., have an advertisement of a new battery in this issue of THE JOURNAL, to which the attention of our readers is directed. They claim for this battery superiority over other batteries of the kind. See the advertisement and write for circulars.

GEORGE K. HOPKINS, of St. Louis, offers something new in this issue in the way of saddle-bags and buggy-cases. We have examined them and are confident that they are superior to any saddle-bags or buggy-cases on the market.

THE MALTINE MANUFACTURING CO., of New York, have something of interest to say to our readers in a new advertisement to be found in this number of THE JOURNAL. They will gladly send samples of any of their goods to physicians who will pay express charges. Read the advertisement and write them for what you want.

REED & CARNRICK, NEW YORK.—This firm and the goods they manufacture are too well known to require any words of commendation from us. Their preparations are staple. Write them for any information you do not get from the advertisement and they will cheerfully furnish it.

